# Dynamical Analysis of an Improved Bidirectional Immunization SIR Model in Complex Network

**DOI:** 10.3390/e26030227

**Published:** 2024-03-02

**Authors:** Shixiang Han, Guanghui Yan, Huayan Pei, Wenwen Chang

**Affiliations:** 1 School of Electronic and Information Engineering, Lanzhou Jiaotong University, Lanzhou 730070, China; hsx961002@126.com (S.H.); pei123com@126.com (H.P.); changwenwen11@mails.ucas.ac.cn (W.C.); 2 Key Laboratory of Media Convergence Technology and Communication, Lanzhou 730070, China

**Keywords:** SIR epidemic model, complex network, continuous-time Markov chain, basic reproduction number

## Abstract

In order to investigate the impact of two immunization strategies—vaccination targeting susceptible individuals to reduce their infection rate and clinical medical interventions targeting infected individuals to enhance their recovery rate—on the spread of infectious diseases in complex networks, this study proposes a bilinear SIR infectious disease model that considers bidirectional immunization. By analyzing the conditions for the existence of endemic equilibrium points, we derive the basic reproduction numbers and outbreak thresholds for both homogeneous and heterogeneous networks. The epidemic model is then reconstructed and extensively analyzed using continuous-time Markov chain (CTMC) methods. This analysis includes the investigation of transition probabilities, transition rate matrices, steady-state distributions, and the transition probability matrix based on the embedded chain. In numerical simulations, a notable concordance exists between the outcomes of CTMC and mean-field (MF) simulations, thereby substantiating the efficacy of the CTMC model. Moreover, the CTMC-based model adeptly captures the inherent stochastic fluctuation in the disease transmission, which is consistent with the mathematical properties of Markov chains. We further analyze the relationship between the system’s steady-state infection density and the immunization rate through MCS. The results suggest that the infection density decreases with an increase in the immunization rate among susceptible individuals. The current research results will enhance our understanding of infectious disease transmission patterns in real-world scenarios, providing valuable theoretical insights for the development of epidemic prevention and control strategies.

## 1. Introduction

The mathematical modeling of infectious diseases is of significant importance, as it facilitates a profound understanding of disease transmission patterns and aids in formulating corresponding strategies for epidemic prevention. Beginning with the pioneering work of Kermack and McKendrick [1], mathematical modeling has demonstrated its effectiveness as a powerful tool for understanding the transmission dynamics of infectious diseases. The classical compartmental model they introduced categorizes the population within an epidemic area into three groups: susceptible, infected, and recovered individuals. Additionally, this model also elucidates the transmission dynamics of the disease across the population through a system of differential equations grounded in mean-field methods. Indeed, the compartmental model has gained broad acceptance and is widely utilized to explore the intricacies of infectious disease dynamics, maintaining its significance in the contemporary era. Later, in 1932, Kermack and McKendrick [2] introduced the SIS compartmental model. Through a theoretical analysis of this model, they also developed the threshold theory to distinguish the prevalence of infectious diseases, laying the foundation for exploring epidemic dynamics.

Following that, infectious disease models have become increasingly complex, incorporating factors such as age structures, specific disease attributes, and time-delay effects. This enhancement allows these models to more accurately depict intricate real-world scenarios [3,4,5,6,7,8]. However, traditional epidemic models assume homogeneous mixing of the population, with equal opportunities for contact among individuals, thus neglecting individual heterogeneity. The advent of complex networks provides a fresh perspective for constructing propagation models that can capture individual-level heterogeneity [9,10,11]. This advancement further enhances the alignment of infectious disease dynamics research with real-world scenarios. In recent years, an increasing amount of research has been directed towards understanding the impact of population contact networks and their heterogeneity on the transmission of infectious diseases [12,13,14,15,16,17,18,19,20,21]. In particular, Huo et al. [22] introduced a fractional SIR model with birth and death rates on heterogeneous complex networks and analyzed both its local and global stability of disease-free and endemic equilibrium. Additionally, Chen et al. examined an SIRS epidemic model with vaccination on heterogeneous networks [23]. In their study, the investigation of the global stability of both the disease-free and endemic equilibrium points is carried out by constructing suitable Lyapunov functions. This line of inquiry can be traced back to 2001, when Pastor and Vespignani introduced mean-field-based epidemic models within heterogeneous networks [9].

On the other hand, additional researchers have broadened the scope of infectious disease dynamics research by investigating factors such as heterogeneous infection rates, vaccination strategies, and immune coverage. In particular, in 2013, Cai et al. [24] proposed an enhanced susceptible–vaccinated–infected–recovered (SVIR) epidemic model that incorporates a diversity of infection rate of the individuals. This study found that the heterogeneity in infection rates can either hinder or accelerate the spread of the epidemic, depending on the number of vaccinated individuals introduced into the population and the patterns of contact among individuals. Subsequently, Cai et al. [25] further investigated the impact of vaccination on the spread of infectious diseases based on the SVIR model on complex networks. In this study, the authors conducted comparative analyses through experimental simulations to evaluate the immunization effects of vaccination methods such as pure vaccination/continuous vaccination and continuous vaccination with random mutation.

Furthermore, in addition to mean-field-based stochastic differential equations, epidemic models constructed using the continuous-time Markov chain (CTMC) method offer a probabilistic perspective to comprehend disease transmission patterns within a population [26,27]. In the mathematical modeling of infectious diseases, CTMC models offer a more accurate portrayal of the ongoing stochastic fluctuations inherent in the spread of diseases within population contact networks, surpassing conventional mean-field models in this regard. Additionally, in recent years, a microscopic Markov-chain approach (MMCA) has also been employed to depict the spread of epidemics. In 2011, Gómez et al. [28] restructured the discrete-time SIS epidemic model using MMCA and observed that the epidemic prevalence obtained through this approach is consistent with that obtained through simulations. Subsequently, in 2014, Cai et al. [29] expanded the effective degree Markov-chain approach, initially designed for analyzing continuous-time epidemic processes, to encompass discrete-time SIS or SIR epidemic processes on uncorrelated complex networks. In numerical simulations, it is demonstrated that the final epidemic size and the time series of infected individuals obtained from MMCA closely match those produced by Monte Carlo simulations (MCSs). In summary, the introduction of probabilistic methods, exemplified by Markov chains, has injected fresh vitality into the field of infectious disease dynamics research, offering a novel perspective for further exploration.

In this research, we introduce an improved network-based SIR epidemic model that integrates bidirectional immunization rates and accounts for the birth and death rates of individuals. In the theoretical analysis, we first calculate the basic reproduction number for both homogeneous and heterogeneous networks by analyzing the existence of the endemic equilibrium. Subsequently, we restructure our mean-field epidemic model using the CTMC method. Consequently, we further analyze the transition probabilities, transition rate matrix, steady-state distribution, and transition probability matrix based on the embedded chain of the CTMC epidemic model. In numerical simulations, we utilize the Gillespie algorithm to depict the CTMC-based epidemic model. We then compare the evolution patterns of our proposed model using three different simulation methods: mean-field method (MF), Monte Carlo simulation (MCS), and CTMC. The simulation results demonstrate that, compared to MF and MCS, CTMC demonstrates finer details of the evolution of infectious diseases over shorter time intervals, thereby more accurately characterizing the evolutionary patterns of our model. Notably, this approach employs probabilistic methods to describe the randomness of infectious disease transmission, offering new insights into the study of infectious disease dynamics.

In Section 2, we thoroughly conduct a theoretical analysis of the proposed epidemic model using both mean-field and continuous-time Markov chain approaches. This analysis encompasses the derivation of the basic reproduction numbers, steady-state distributions, and transition probabilities for epidemic spreading on both homogeneous and heterogeneous networks. The results from the simulations and the subsequent discussion are detailed in Section 3. A summary of the findings is provided in Section 4.

## 2. Model

In this study, we integrate immunization measures targeting susceptible individuals and clinical treatment measures aimed at infected individuals into the traditional SIR network-based infectious disease model. Furthermore, our proposed epidemic model accounts for the birth and death rates of individuals. In our model, akin to the standard SIR model, S(t), I(t), and R(t) denote the proportions of susceptible, infected, and recovered individuals, respectively, at each time step. Additionally, we employ the notations β and γ to symbolize the infection and recovery rates for susceptible and infected individuals, respectively. In addition, deviating from conventional propagation models, we further integrate the birth rate *b* and death rate *d* into our model to capture the intricate dynamics of population evolution. In particular, it should be mentioned that the death rate *d* is the natural death rate, not one related to the epidemic. Furthermore, parameters δ and λ are also introduced in this model, representing the reduction rate of infected individuals and the increase rate of recovered individuals, respectively, after implementing the corresponding immunization and treatment measures. Considering the practical significance of δ and λ in clinical immunization measures, δ and λ can respectively be regarded as the effective vaccination rate of susceptible individuals and the immunity rate of individuals recovered from natural infection. Additionally, $\frac{dS(t)}{dt}$, $\frac{dI(t)}{dt}$, and $\frac{dR(t)}{dt}$ are used to represent the instantaneous rates of change of susceptible, infected, and recovered individuals with respect to time step *t*.

### 2.1. Mean-Field Equations and Analysis

According to the propagation rules proposed in this study, the mean-field differential equations for our model in homogeneous networks can be expressed as follows:

(1) $$\begin{array}{c} \left\{\begin{array}{c} \begin{array}{c} \frac{dS(t)}{dt}=bN-d\cdot S\left(t\right)-\beta \left(1-\delta \right)\langle k\rangle S\left(t\right)I\left(t\right), \end{array}\;\\ \begin{array}{c} \frac{dI(t)}{dt}=\beta \left(1-\delta \right)\langle k\rangle S\left(t\right)I\left(t\right)-\gamma \left(1+\lambda \right)I\left(t\right)-d\cdot I\left(t\right), \end{array}\;\\ \begin{array}{c} \frac{dR(t)}{dt}=\gamma \left(1+\lambda \right)I\left(t\right)-d\cdot R\left(t\right), \end{array}\; \end{array}\right. \end{array}$$

where $\langle k\rangle =\sum_{k}kp(k)$ represents the average degree of networks, with p(k) denoting the degree distribution of each individual in the networks. This indicates that, in homogeneous networks, the number of contacts between any individual node and other nodes can be approximated by the average degree $\langle k\rangle$. Additionally, when considering the density of susceptible, infected, or recovered individuals, *N* is commonly assumed to be equal to 1, representing the normalization of the population size in the context of the model.

Furthermore, in real-world human contact networks, individuals exhibit a significant degree of heterogeneity. Therefore, to provide a more comprehensive understanding of epidemic spreading dynamics on heterogeneous networks, Pastor-Satorras and Vespignani introduced mean-field equations specifically tailored for such networks [9]. Similarly, within the context of this study, the densities of susceptible, infected, and recovered individuals associated with degree *k* are denoted as $S_{k}$, $I_{k}$, and $R_{k}$, respectively. Therefore, based on the propagation rules proposed in this paper, the mean-field equations for heterogeneous networks can be denoted as follows:

(2) $$\begin{array}{c} \left\{\begin{array}{c} \begin{array}{c} \frac{dS_{k}}{dt}=bN-d\cdot S_{k}-\beta \left(1-\delta \right)kS_{k}\Theta_{k}, \end{array}\;\\ \begin{array}{c} \frac{dI_{k}}{dt}=\beta \left(1-\delta \right)kS_{k}\Theta_{k}-\gamma \left(1+\lambda \right)I_{k}-d\cdot I_{k}, \end{array}\;\\ \begin{array}{c} \frac{dR_{k}}{dt}=\gamma \left(1+\lambda \right)I_{k}-d\cdot R_{k}, \end{array}\; \end{array}\right. \end{array}$$

where $\Theta_{k}=\frac{1}{\langle k\rangle }\sum_{k}kp\left(k\right)I_{k}$ signifies the fraction of infected edges within the network, reflecting the likelihood that each edge connecting a susceptible individual is linked to an infected individual. Continuing our analysis, we further investigate the equilibrium solution of the epidemic model in heterogeneous networks [30]. Firstly, by setting $\frac{dS_{k}}{dt}=\frac{dI_{k}}{dt}=I_{k}=0$, we can derive the disease-free solution of Equation (2) depicted as follows:

(3) $$\begin{array}{c} E_{0}\left(S_{k}^{0},I_{k}^{0},R_{k}^{0}\right)=\left(\frac{bN}{d},0,0\right),\;k=1,2,\cdots ,N, \end{array}$$

that is, the disease-free equilibrium point always exists in the system (2). Furthermore, it is necessary to discuss the endemic equilibrium solution of this model.

Similar to the disease-free equilibrium solution, setting $\frac{dS_{k}}{dt}=\frac{dI_{k}}{dt}=0$, the endemic equilibrium solution can be expressed as follows:

(4) $$\begin{array}{c} \left\{\begin{array}{c} S_{k}^{*}=\frac{bN}{d+\beta k\left(1-\delta \right)\Theta_{k}},\;\\ I_{k}^{*}=\frac{\beta k\left(1-\delta \right)S_{k}\Theta_{k}}{\gamma (1+\lambda )+d}. \end{array}\right. \end{array}$$

From Equation (4), it is obvious that $I_{k}^{*}$ is a function that includes the independent variable $S_{k}$; therefore, substituting $S_{k}^{*}$ into $I_{k}^{*}$, the result is as follows:

(5) $$\begin{array}{c} I_{k}^{*}=\frac{bN\beta k\Theta_{k}\left(1-\delta \right)}{\left[d+\beta k\Theta_{k}\left(1-\delta \right)\right]\left[\gamma \left(1+\lambda \right)+d\right]}. \end{array}$$

Similarly, $\Theta_{k}$ is an expression containing the argument $I_{k}$. Substituting Equation (5) into $\Theta_{k}$, the self-consistent equation for $\Theta_{k}$ is obtained as follows:

(6) $$\begin{array}{c} \Theta_{k}=\sum_{k}\frac{kp\left(k\right)bN\beta k\left(1-\delta \right)\Theta_{k}}{\langle k\rangle \left[d+\beta k\left(1-\delta \right)\Theta_{k}\right]\left[\gamma \left(1+\lambda \right)+d\right]}. \end{array}$$

It is easy to obtain that $\Theta_{k}=0$ is a trivial solution of Equation (6), corresponding to the disease-free equilibrium point $E_{0}$. Therefore, when there is a nontrivial solution $\Theta_{k}\in \left(0,1\right)$, this model can be considered to have an endemic equilibrium point $E_{*}$. Thus, we further discuss the existence of a non-zero solution to $\Theta_{k}$. Firstly, construct the auxiliary function $F(\Theta_{k})$ as follows:

(7) $$\begin{array}{c} F\left(\Theta_{k}\right)=\Theta_{k}-\frac{1}{\langle k\rangle }\sum_{k}\frac{bN\beta \left(1-\delta \right)k^{2}p\left(k\right)\Theta_{k}}{\left[d+\beta k\left(1-\delta \right)\Theta_{k}\right]\left[\gamma \left(1+\lambda \right)+d\right]}. \end{array}$$

Obviously, if there is a non-zero solution for $F(\Theta_{k})=0$, it indicates that our model satisfies the conditions for an epidemic outbreak, thus confirming the existence of an endemic equilibrium point. Therefore, to derive the condition for the existence of a non-zero solution for $F(\Theta_{k})=0$, we will further explore the derivative of $F(\Theta_{k})$. The first and second derivatives of $F(\Theta_{k})$ are given as follows:

(8) $$\begin{array}{c} \frac{dF(\Theta_{k})}{d\Theta_{k}}=1-\frac{1}{\langle k\rangle }\sum_{k}\frac{bdN\beta \left(1-\delta \right)k^{2}p\left(k\right)}{{\left[d+\beta k\left(1-\delta \right)\Theta_{k}\right]}^{2}\left[\gamma (1+\lambda )+d\right]}, \end{array}$$

(9) $$\begin{array}{c} \frac{d^{2}F\left(\Theta_{k}\right)}{d{\Theta_{k}}^{2}}=\frac{2}{\langle k\rangle }\sum_{k}\frac{bdN\beta^{2}{\left(1-\delta \right)}^{2}k^{3}p\left(k\right)}{{\left[d+\beta k\left(1-\delta \right)\Theta_{k}\right]}^{3}\left[\gamma (1+\lambda )+d\right]}. \end{array}$$

From Equation (9), it is evident that $\frac{d^{2}F\left(\Theta_{k}\right)}{d{\Theta_{k}}^{2}}>0$, indicating that $F(\Theta_{k})$ is a concave function. In addition, from Equation (6), we can deduce that F(0)=0. Next, we examine the value of the other endpoint F(1). The result of F(1) can be denoted as follows:

(10) $$\begin{array}{c} F\left(1\right)=1-\frac{1}{\langle k\rangle }\sum_{k}\frac{bN\beta \left(1-\delta \right)k^{2}p\left(k\right)}{\left[d+\beta k(1-\delta )\right]\left[\gamma (1+\lambda )+d\right]}. \end{array}$$

It is clear that $\frac{bN\beta (1-\delta )}{\gamma (1+\lambda )+d}<\beta \left(1-\delta \right)\Rightarrow \frac{bN\beta k(1-\delta )}{\gamma (1+\lambda )+d}<d+\beta k\left(1-\delta \right)$, resulting in the following inequality:

(11) $$\begin{array}{c} F\left(1\right)>1-\frac{1}{\langle k\rangle }\sum_{k}kp(k)=0. \end{array}$$

Based on the aforementioned analysis, it can be concluded that the necessary and sufficient condition for $F(\Theta_{k})=0$ to have a nontrivial solution at $\Theta_{k}\neq 0$ is ${\left.\frac{dF(\theta_{k})}{d\theta_{k}}\right|}_{\theta_{k}=0}<0$, which can be obtained as follows:

(12) $$\begin{array}{c} 1-\frac{\langle k^{2}\rangle }{\langle k\rangle }\frac{bN\beta (1-\delta )}{d\left[d+\gamma (1+\lambda )\right]}<0. \end{array}$$

Based on Equation (12), it can be observed that, when $\frac{\langle k^{2}\rangle }{\langle k\rangle }\frac{bN\beta (1-\delta )}{d\left[d+\gamma (1+\lambda )\right]}>1$, system (2) demonstrates an endemic equilibrium point, which signifies the occurrence of an epidemic outbreak. Therefore, according to the definition, the basic reproduction number of this model in heterogeneous networks can be characterized as follows:

(13) $$\begin{array}{c} {\hat{R}}_{0}=\frac{bN\beta (1-\delta )}{d^{2}+\gamma d\left(1+\lambda \right)}\frac{\langle k^{2}\rangle }{\langle k\rangle }. \end{array}$$

Thus, when ${\hat{R}}_{0}=1$, the epidemic in heterogeneous networks is in a critical state of outbreak. Therefore, in accordance with the definition of the epidemic outbreak threshold, the outbreak threshold of our model in heterogeneous networks can be represented as follows:

(14) $$\begin{array}{c} {\hat{\beta }}_{c}=\frac{d^{2}+\gamma d\left(1+\lambda \right)}{bN(1-\delta )}\frac{\langle k\rangle }{\langle k^{2}\rangle }. \end{array}$$

This means that, when the infection rate β exceeds $\frac{d^{2}+\gamma d\left(1+\lambda \right)}{bN(1-\delta )}\frac{\langle k\rangle }{\langle k^{2}\rangle }$, an outbreak of infectious diseases will occur in heterogeneous networks.

Specifically, in a homogeneous network with a degree distribution $p\left(k\right)∼\frac{\xi^{k}e^{-\xi }}{k!}$ following a Poisson distribution, where ξ is the parameter, the approximate equality $\frac{\langle k^{2}\rangle }{\langle k\rangle }\approx \langle k\rangle$ holds. This indicates that the basic reproduction number in homogeneous networks can be expressed as follows:

(15) $$\begin{array}{c} {\tilde{R}}_{0}=\frac{bN\beta \left(1-\delta \right)\langle k\rangle }{d^{2}+\gamma d\left(1+\lambda \right)}. \end{array}$$

Similarly, the outbreak threshold of infectious diseases in homogeneous networks can be obtained as follows:

(16) $$\begin{array}{c} {\tilde{\beta }}_{c}=\frac{d^{2}+\gamma d\left(1+\lambda \right)}{bN\left(1-\delta \right)\langle k\rangle }. \end{array}$$

### 2.2. Modeling and Analysis Based on Continuous-Time Markov Chain

In this section, we utilize the continuous-time Markov chain (CTMC) to formulate the proposed epidemic model and derive the steady-state distribution, as well as the state transition probabilities, of the CTMC-based propagation model [26]. Initially, it is evident that the array consisting of the susceptible node density S(t) and the infected node density I(t) at any time *t* can be represented as a CTMC. In addition, the discrete random variables in the CTMC-based SIR model adhere to the following:

(17) $$\begin{array}{c} S(t),I(t)\in \{0,1,2,\cdots ,N\}, \end{array}$$

where $t\in \left[0,\infty \right)$. Differently from the previous analysis of the mean-field SIR model, S(t) and I(t) in Markov chains represent the number of susceptible and infected individuals in the system at time step *t*, respectively. Moreover, any increase in I(t) within a certain time interval is inevitably accompanied by an equivalent decrease in S(t) during the same period. In addition, *s* and *i* in lowercase represent the discrete random variable values from the set {0,1,2,⋯,N}. Thus, when the network contains *i* infected nodes, the transition probability for the stochastic process over a short time interval of Δt>0 can be represented as follows:

(18) $$\begin{array}{c} p_{\left(s,i),(s+k,i+j\right)}\left(\Delta t\right)=P\left(S\left(t+\Delta t\right),I\left(t+\Delta t\right)\right)=\left(s+k,i+j\right)\left|\left(S(t),I(t))=(s,i\right)\right.. \end{array}$$

According to the properties of CTMC, we assume that, within a sufficiently small time interval Δt, the network can only experience one of the following events: an increase by one infected node, a decrease by one infected node, or the number of infected nodes remains unchanged. Therefore, it is clear that (k,j)∈{(−1,+1),(0,−1),(0,0)}. The transition probabilities of these three cases will be discussed separately. When $\left(k,j\right)=\left(-1,+1\right)$, as per the proposed propagation model in this paper, the transition probability of the CTMC in homogeneous networks can be expressed as follows:

(19) $$\begin{array}{c} {\tilde{p}}_{\left(s,i),(s-1,i+1\right)}\left(\Delta t\right)=\frac{s}{N}\beta i\left(1-\delta \right)\langle k\rangle \Delta t≜{\tilde{\rho }}_{\left(s,i\right)}\Delta t, \end{array}$$

which can be defined as ${\tilde{\rho }}_{\left(s,i\right)}\Delta t$. Similarly, the transition probabilities for reducing one infected node and maintaining the same number of infected nodes in homogeneous networks can be respectively denoted as follows:

(20) $$\begin{array}{c} {\tilde{p}}_{\left(s,i),(s,i-1\right)}\left(\Delta t\right)=\gamma i\left(1+\lambda \right)\Delta t\overset{\Delta }{=}{\tilde{\mu }}_{\left(s,i\right)}\Delta t, \end{array}$$

(21) $$\begin{array}{c} {\tilde{p}}_{\left(s,i),(s,i\right)}\left(\Delta t\right)=1-\left(\frac{s}{N}\beta i\left(1-\delta \right)\langle k\rangle +\gamma i\left(1+\lambda \right)\right)\Delta t\overset{\Delta }{=}1-\left({\tilde{\rho }}_{\left(s,i\right)}+{\tilde{\mu }}_{\left(s,i\right)}\right)\Delta t, \end{array}$$

which can be represented as ${\tilde{\mu }}_{\left(s,i\right)}\Delta t$ and $1-({\tilde{\rho }}_{\left(s,i\right)}+{\tilde{\mu }}_{\left(s,i\right)})\Delta t$. Similarly, in heterogeneous networks, the transition probabilities of increasing an infected node, decreasing an infected node, and maintaining the number of infected nodes can be gained as follows:

(22) $$\begin{array}{c} {\hat{p}}_{\left(s,i),(s-1,i+1\right)}\left(\Delta t\right)=\frac{s}{N}\beta i\left(1-\delta \right)\frac{\langle k^{2}\rangle }{\langle k\rangle }\Delta t\overset{\Delta }{=}{\hat{\rho }}_{\left(s,i\right)}\Delta t, \end{array}$$

(23) $$\begin{array}{c} {\hat{p}}_{\left(s,i),(s,i-1\right)}\left(\Delta t\right)=\gamma i\left(1+\lambda \right)\Delta t\overset{\Delta }{=}{\hat{\mu }}_{\left(s,i\right)}\Delta t, \end{array}$$

(24) $$\begin{array}{c} {\hat{p}}_{\left(s,i),(s,i\right)}\left(\Delta t\right)=1-\left(\frac{s}{N}\beta i\left(1-\delta \right)\frac{\langle k^{2}\rangle }{\langle k\rangle }+\gamma i\left(1+\lambda \right)\right)\Delta t\overset{\Delta }{=}1-\left({\hat{\rho }}_{\left(s,i\right)}+{\hat{\mu }}_{\left(s,i\right)}\right)\Delta t. \end{array}$$

Building upon the aforementioned equations, we can proceed to derive the transition probabilities matrix for the states (S(t),I(t)), denoted as P, as follows:

(25) $$\begin{array}{c} P=\left[\begin{array}{cccccc} 1-\rho_{\left(s,1\right)}\Delta t & \rho_{\left(s,1\right)}\Delta t & 0 & 0 & \cdots & 0\\ \mu_{\left(s,2\right)}\Delta t & 1-(\rho_{\left(s-1,2\right)}\Delta t+\mu_{\left(s,2\right)}\Delta t) & \rho_{\left(s-1,2\right)}\Delta t & 0 & \cdots & 0\\ 0 & \mu_{\left(s,3\right)}\Delta t & 1-(\rho_{\left(s-2,3\right)}\Delta t+\mu_{\left(s,3\right)}\Delta t) & \rho_{\left(s-2,3\right)}\Delta t & \cdots & 0\\ \vdots & \vdots & \vdots & \vdots & \ddots & \vdots \\ 0 & 0 & 0 & 0 & \cdots & \rho_{\left(s-N+2,N-1\right)}\Delta t\\ 0 & 0 & 0 & 0 & \cdots & 1-\mu_{\left(s,N\right)}\Delta t \end{array}\right]. \end{array}$$

According to matrix P, it is evident that I(0)=1. Moving forward, we further consider the transition rates within our proposed network-based SIR model using a continuous-time Markov chain. Firstly, the rate at which the Markov chain transitions from state (s,i) to state (s+k,i+j) can be represented as follows:

(26) $$\begin{array}{c} q\left(\left(s,i\right),\left(s+k,i+j\right)\right)=\lim_{h\to 0}\frac{p_{\left(s,i),(s+k,i+j\right)}\left(h\right)}{h}, \end{array}$$

where $p_{\left(s,i),(s+k,i+j\right)}\left(h\right)$ represents the probability of transitioning from state (s,i) to state (s+k,i+j) within time *h*. Hence, the transition rates for adding and removing an infected node in homogeneous networks can be described as follows:

(27) $$\begin{array}{c} \tilde{q}\left(\left(s,i\right),\left(s-1,i+1\right)\right)=\lim_{\Delta t\to 0}\frac{{\tilde{p}}_{\left(s,i),(s-1,i+1\right)}\left(\Delta t\right)}{\Delta t}={\tilde{\rho }}_{\left(s,i\right)}=\frac{s}{N}\beta i\left(1-\delta \right)\langle k\rangle , \end{array}$$

(28) $$\begin{array}{c} \tilde{q}\left(\left(s,i\right),\left(s,i-1\right)\right)=\lim_{\Delta t\to 0}\frac{{\tilde{p}}_{\left(s,i),(s,i-1\right)}\left(\Delta t\right)}{\Delta t}={\tilde{\mu }}_{\left(s,i\right)}=\gamma i\left(1+\lambda \right). \end{array}$$

Similarly, the transition rates for the addition and removal of an infected node in heterogeneous networks can be obtained as follows:

(29) $$\begin{array}{c} \hat{q}\left(\left(s,i\right),\left(s-1,i+1\right)\right)=\lim_{\Delta t\to 0}\frac{{\hat{p}}_{\left(s,i),(s-1,i+1\right)}\left(\Delta t\right)}{\Delta t}={\hat{\rho }}_{\left(s,i\right)}=\frac{s}{N}\beta i\left(1-\delta \right)\frac{\langle k^{2}\rangle }{\langle k\rangle }, \end{array}$$

(30) $$\begin{array}{c} \hat{q}\left(\left(s,i\right),\left(s,i-1\right)\right)=\lim_{\Delta t\to 0}\frac{{\hat{p}}_{\left(s,i),(s,i-1\right)}\left(\Delta t\right)}{\Delta t}={\hat{\mu }}_{\left(s,i\right)}=\gamma i\left(1+\lambda \right). \end{array}$$

Furthermore, according to the following properties of the CTMC model

(31) $$\begin{array}{c} \left\{\begin{array}{c} q((s,i),(s,i))\leq 0,\;\\ q((s,i),(s+k,i+j))\geq 0((k,j)\neq (0,0)),\;\\ \sum_{\left(k,j\right)}q((s,i),(s+k,i+j))=0, \end{array}\right. \end{array}$$

the transition rates matrix of the proposed CTMC-based SIR epidemic model can be acquired as follows:

(32) $$\begin{array}{c} Q=\left[\begin{array}{cccccc} -\rho_{\left(s,1\right)} & \rho_{\left(s,1\right)} & 0 & 0 & \cdots & 0\\ \mu_{\left(s,2\right)} & -(\rho_{\left(s-1,2\right)}+\mu_{\left(s,2\right)}) & \rho_{\left(s-1,2\right)} & 0 & \cdots & 0\\ 0 & \mu_{\left(s,3\right)} & -(\rho_{\left(s-2,3\right)}+\mu_{\left(s,3\right)}) & \rho_{\left(s-2,3\right)} & \cdots & 0\\ \vdots & \vdots & \vdots & \vdots & \ddots & \vdots \\ 0 & 0 & 0 & 0 & \cdots & \rho_{\left(s-N+2,N-1\right)}\\ 0 & 0 & 0 & 0 & \cdots & -\mu_{\left(s,N\right)} \end{array}\right]. \end{array}$$

Based on matrix (32), the state transitions in this model only occur between adjacent states or remain unchanged. Therefore, this chain can be regarded as a birth and death process within CTMC. Therefore, for a birth and death process, it satisfies the following local balance equation:

(33) $$\begin{array}{c} \pi_{i}q\left(\left(s,i\right),\left(s+k,i+j\right)\right)=\pi_{i+j}q\left(\left(s+k,i+j\right),\left(s,i\right)\right), \end{array}$$

where $\pi_{i+j}=\lim_{t\to \infty }p_{\left(s,i),(s+k,i+j\right)}\left(t\right)$ denotes the probability of the network containing i+j infected nodes in the steady state. Therefore, based on Equation (31), we can derive the following system of local balance equations for our model:

(34) $$\begin{array}{c} \left\{\begin{array}{c} \rho_{\left(s,1\right)}\pi_{1}=\mu_{\left(s,2\right)}\pi_{2},\\ \rho_{\left(s-1,2\right)}\pi_{2}=\mu_{\left(s,3\right)}\pi_{3},\\ \vdots \\ \rho_{\left(s-N+2,N-1\right)}\pi_{N-1}=\mu_{\left(s,N\right)}\pi_{N},\\ \pi_{1}+\pi_{2}+\cdots +\pi_{N-1}+\pi_{N}=1. \end{array}\right. \end{array}$$

According to Equation (34), a recursive formula for $\pi_{i}$ can be derived as follows:

(35) $$\begin{array}{c} \frac{\pi_{i+1}}{\pi_{i}}=\frac{\rho_{\left(s-i+1,i\right)}}{\mu_{\left(s,i+1\right)}}. \end{array}$$

Following that, we can further express the recursive formulas for the steady-state distribution of the number of infected nodes *i* in both homogeneous and heterogeneous networks as follows:

(36) $$\begin{array}{c} \frac{{\tilde{\pi }}_{i+1}}{{\tilde{\pi }}_{i}}=\frac{{\tilde{\rho }}_{\left(s-i+1,i\right)}}{{\tilde{\mu }}_{\left(s,i+1\right)}}=\frac{\left(s-i+1\right)\beta i\left(1-\delta \right)\langle k\rangle }{N\gamma (i+1)(1+\lambda )}, \end{array}$$

(37) $$\begin{array}{c} \frac{{\hat{\pi }}_{i+1}}{{\hat{\pi }}_{i}}=\frac{{\hat{\rho }}_{\left(s-i+1,i\right)}}{{\hat{\mu }}_{\left(s,i+1\right)}}=\frac{\left(s-i+1\right)\beta i\left(1-\delta \right)\langle k^{2}\rangle }{N\gamma \left(i+1\right)\left(1+\lambda \right)\langle k\rangle }. \end{array}$$

Thus, based on the equations above, the steady-state probability of $\pi_{i}$ can be easily obtained as follows:

(38) $$\begin{array}{c} \pi_{i}=\frac{\rho_{\left(s-i+2,i-1\right)}\rho_{\left(s-i+3,i-2\right)}\cdots \rho_{\left(s,1\right)}}{\mu_{\left(s,i\right)}\mu_{\left(s,i-1\right)}\cdots \mu_{\left(s,2\right)}}\pi_{1}, \end{array}$$

which represents the probability that the system contains *i* infected nodes when it reaches steady state. In addition, according to the property of $\sum_{i=1}^{N}\pi_{i}=1$, $\pi_{1}$ can be expressed as follows:

(39) $$\begin{array}{c} \pi_{1}=\frac{1}{\sum_{i=1}^{N}\frac{\rho_{\left(s-i+2,i-1\right)}\rho_{\left(s-i+3,i-2\right)}\cdots \rho_{\left(s,1\right)}}{\mu_{\left(s,i\right)}\mu_{\left(s,i-1\right)}\cdots \mu_{\left(s,2\right)}}}. \end{array}$$

Substituting Equation (39) into Equation (38), the complete expression of $\pi_{i}$ can be represented as follows:

(40) $$\begin{array}{c} \pi_{i}=\frac{\rho_{\left(s-i+2,i-1\right)}\rho_{\left(s-i+3,i-2\right)}\cdots \rho_{\left(s,1\right)}}{\mu_{\left(s,i\right)}\mu_{\left(s,i-1\right)}\cdots \mu_{\left(s,2\right)}\sum_{i=1}^{N}\frac{\rho_{\left(s-i+2,i-1\right)}\rho_{\left(s-i+3,i-2\right)}\cdots \rho_{\left(s,1\right)}}{\mu_{\left(s,i\right)}\mu_{\left(s,i-1\right)}\cdots \mu_{\left(s,2\right)}}}. \end{array}$$

Hence, the steady-state probability distribution of containing *i* infected nodes in the system can be denoted respectively in homogeneous and heterogeneous network models as follows:

(41) $$\begin{array}{c} {\tilde{\pi }}_{i}=\frac{s!{\left[\beta \left(1-\delta \right)\langle k\rangle \right]}^{i-1}}{i\left(s-i+1\right)!{\left[N\gamma (1+\lambda )\right]}^{i-1}\sum_{i=1}^{N}\frac{s!}{i(s-i+1)!}{\left[\frac{\beta \left(1-\delta \right)\langle k\rangle }{N\gamma (1+\lambda )}\right]}^{i-1}}, \end{array}$$

(42) $$\begin{array}{c} {\hat{\pi }}_{i}=\frac{s!{\left[\beta \left(1-\delta \right)\langle k^{2}\rangle \right]}^{i-1}}{i\left(s-i+1\right)!{\left[N\gamma \left(1+\lambda \right)\langle k\rangle \right]}^{i-1}\sum_{i=1}^{N}\frac{s!}{i(s-i+1)!}{\left[\frac{\beta \left(1-\delta \right)\langle k^{2}\rangle }{N\gamma \left(1+\lambda \right)\langle k\rangle }\right]}^{i-1}}. \end{array}$$

Consequently, we further consider the embedded Markov chain of our model. For a continuous-time Markov chain with the transition rate matrix Q, the set of $r_{\left(s,i),(s+k,i+j\right)}$ that satisfies the following condition constitutes the corresponding embedded chain.

(43) $$\begin{array}{c} r_{\left(s,i),(s+k,i+j\right)}=\left\{\begin{array}{c} \frac{q_{\left(s,i),(s+k,i+j\right)}}{\sum_{\left(k,j)\neq (0,0\right)}q_{\left(s,i),(s+k,i+j\right)}}=\frac{q_{\left(s,i)(s+k,i+j\right)}}{q_{\left(s,i\right)}},\left(k,j\right)\neq \left(0,0\right),\;\\ 0,(k,j)=(0,0), \end{array}\right. \end{array}$$

where $q_{\left(s,i),(s+k,i+j\right)}$ represents the corresponding elements of the matrix Q and $q_{\left(s,i\right)}$ is the rate of leaving state (s,i). Additionally, it is important to note that $q_{\left(s,i\right)}=-q_{\left(s,i),(s,i\right)}$. Therefore, compared with the birth and death process, the embedded chain neglects the case where the state remains unchanged. Subsequently, let $T_{\left(s-1,i+1\right)}$ and $T_{\left(s,i-1\right)}$ denote the durations that the Markov chain spends in state (s,i) before transitioning to state (s−1,i+1) and (s,i−1), respectively. According to the properties of CTMC, $T_{\left(s-1,i+1\right)}$ and $T_{\left(s,i-1\right)}$ follow exponential distributions with parameters $\rho_{\left(s,i\right)}$ and $\mu_{\left(s,i\right)}$, correspondingly. That is, $T_{\left(s-1,i+1\right)}∼\varepsilon \left(\rho_{\left(s,i\right)}\right)$ and $T_{\left(s,i-1\right)}∼\varepsilon \left(\mu_{\left(s,i\right)}\right)$, where $\varepsilon \left(\rho_{\left(s,i\right)}\right)=\rho_{\left(s,i\right)}e^{-\rho_{\left(s,i\right)}t}$ and $\varepsilon \left(\mu_{\left(s,i\right)}\right)=\mu_{\left(s,i\right)}e^{-\mu_{\left(s,i\right)}t}$. Consequently, grounded in the characteristics of exponential distributions, the transition probability of the embedded chain can be represented as follows:

(44) $$\begin{array}{c} P\left\{X_{t}=\left(s-1,i+1\right)\left|X_{0}=\left(s,t\right)\right.\right\}=P\left\{T_{\left(s-1,i+1\right)}=\min\left(T_{\left(s-1,i+1\right)},T_{\left(s,i-1\right)}\right)\right\}=\frac{\rho_{\left(s,i\right)}}{\rho_{\left(s,i\right)}+\mu_{\left(s,i\right)}},i\neq N, \end{array}$$

(45) $$\begin{array}{c} P\left\{X_{t}=\left(s,i-1\right)\left|X_{0}=\left(s,t\right)\right.\right\}=P\left\{T_{\left(s,i-1\right)}=\min\left(T_{\left(s-1,i+1\right)},T_{\left(s,i-1\right)}\right)\right\}=\frac{\mu_{\left(s,i\right)}}{\rho_{\left(s,i\right)}+\mu_{\left(s,i\right)}},i\neq 0. \end{array}$$

where $X_{t}$ represents the state of the corresponding Markov chain at time step *t*. Therefore, in accordance with the definition of the embedded chain, we can deduce the associated transition probabilities matrix as follows:

(46) $$\begin{array}{c} R=\left[\begin{array}{cccccccc} 0 & 1 & 0 & 0 & \cdots & 0 & 0 & 0\\ \frac{\mu_{\left(s,2\right)}}{\rho_{\left(s-1,2\right)}+\mu_{\left(s,2\right)}} & 0 & \frac{\rho_{\left(s-1,2\right)}}{\rho_{\left(s-1,2\right)}+\mu_{\left(s,2\right)}} & 0 & \cdots & 0 & 0 & 0\\ 0 & \frac{\mu_{\left(s,3\right)}}{\rho_{\left(s-2,3\right)}+\mu_{\left(s,3\right)}} & 0 & \frac{\rho_{\left(s-2,3\right)}}{\rho_{\left(s-2,3\right)}+\mu_{\left(s,3\right)}} & \cdots & 0 & 0 & 0\\ \vdots & \vdots & \vdots & \vdots & \ddots & \vdots & \vdots & \vdots \\ 0 & 0 & 0 & 0 & \cdots & \frac{\mu_{\left(s,N-1\right)}}{\rho_{\left(s-N+2,N-1\right)}+\mu_{\left(s,N-1\right)}} & 0 & \frac{\rho_{\left(s-N+2,N-1\right)}}{\rho_{\left(s-N+2,N-1\right)}+\mu_{\left(s,N-1\right)}}\\ 0 & 0 & 0 & 0 & \cdots & 0 & 1 & 0 \end{array}\right]. \end{array}$$

Additionally, from Equations (44) and (45), it is obvious that

(47) $$\begin{array}{c} P\left\{X_{t}=\left(s-1,i+1\right)\left|X_{0}=\left(s,t\right)\right.\right\}+P\left\{X_{t}=\left(s,i-1\right)\left|X_{0}=\left(s,t\right)\right.\right\}=1, \end{array}$$

which indicates that the normalization condition between probabilities is satisfied. Therefore, by using matrix R, we can obtain the probabilities of the proposed CTMC-based SIR model transitioning between different states at any given time. Furthermore, the main diagonal elements of this matrix are all zeros, indicating that the embedded chain does not account for situations where the number of infected individuals remains unchanged. Following that, according to the properties of the embedded chain, the probabilities of increasing and decreasing an infected node in homogeneous networks at any given time can be represented as follows:

(48) $$\begin{array}{c} \left\{\begin{array}{c} {\tilde{p}}_{\left(s,i),(s-1,i+1\right)}=\frac{{\tilde{\rho }}_{\left(s,i\right)}}{{\tilde{\rho }}_{\left(s,i\right)}+{\tilde{\mu }}_{\left(s,i\right)}}=\frac{\frac{s}{N}\beta i\left(1-\delta \right)\langle k\rangle }{\frac{s}{N}\beta i\left(1-\delta \right)\langle k\rangle +\gamma i\left(1+\lambda \right)},\;\\ {\tilde{p}}_{\left(s,i),(s,i-1\right)}=\frac{{\tilde{\mu }}_{\left(s,i\right)}}{{\tilde{\rho }}_{\left(s,i\right)}+{\tilde{\mu }}_{\left(s,i\right)}}=\frac{\gamma i(1+\lambda )}{\frac{s}{N}\beta i\left(1-\delta \right)\langle k\rangle +\gamma i\left(1+\lambda \right)}. \end{array}\right. \end{array}$$

Similarly, in heterogeneous networks, we can represent the probabilities of adding and removing a single infected node at any given time as follows:

(49) $$\begin{array}{c} \left\{\begin{array}{c} {\hat{p}}_{\left(s,i),(s-1,i+1\right)}=\frac{{\hat{\rho }}_{\left(s,i\right)}}{{\hat{\rho }}_{\left(s,i\right)}+{\hat{\mu }}_{\left(s,i\right)}}=\frac{\frac{s}{N}\beta i\left(1-\delta \right)\frac{\langle k^{2}\rangle }{\langle k\rangle }}{\frac{s}{N}\beta i\left(1-\delta \right)\frac{\langle k^{2}\rangle }{\langle k\rangle }+\gamma i\left(1+\lambda \right)},\;\\ {\hat{p}}_{\left(s,i),(s,i-1\right)}=\frac{{\hat{\mu }}_{\left(s,i\right)}}{{\hat{\rho }}_{\left(s,i\right)}+{\hat{\mu }}_{\left(s,i\right)}}=\frac{\gamma i(1+\lambda )}{\frac{s}{N}\beta i\left(1-\delta \right)\frac{\langle k^{2}\rangle }{\langle k\rangle }+\gamma i\left(1+\lambda \right)}. \end{array}\right. \end{array}$$

Obviously, if i=0, the system reaches an absorbing state and the process of epidemic transmission comes to a halt.

## 3. Numerical Simulations

In 1977, Gillespie introduced a numerical simulation technique tailored for CTMC models, known as either the Gillespie algorithm or the Stochastic Simulation algorithm [31]. In this section, we utilize the mean-field (MF), Monte Carlo simulation (MCS), and Stochastic Simulation (CTMC) methods to simulate an epidemic spreading in our proposed model on both homogeneous and heterogeneous networks. We then compare the results obtained from these three methods. In the study of epidemic dynamics in complex networks, ER random networks and BA scale-free networks are commonly regarded as typical models of homogeneous and heterogeneous networks, respectively [32,33]. In particular, the degree distribution of ER networks follows a Poisson distribution, while the degree distribution of BA networks follows a power-law distribution. Therefore, in the Monte Carlo simulations (MCSs), we employ the ER and BA network models to represent homogeneous and heterogeneous networks. Each of these networks consists of $5\times 10^{3}$ nodes, which is in line with the network sizes used in the MF and CTMC simulations. To validate the practicality of our model, we also simulate the evolutionary process of our model in real network datasets. Moreover, we conduct an in-depth analysis of the impact of bidirectional immunization on the transmission dynamics of infectious diseases within complex networks, utilizing the outcomes derived from Monte Carlo simulations (MCSs). In addition, the impact of the main parameters of our model on the basic reproduction numbers is also discussed in this section.

### 3.1. Impact of Replenishing of Susceptible Individuals on the Model

**Figure 1:**
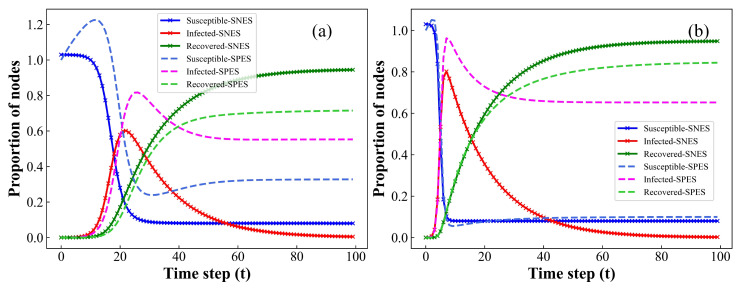
Comparison of the S(t), I(t), and R(t) proportions through mean-field (MF) simulation method with and without the involvement of newly susceptible individuals in the epidemics spreading at each time step *t*. (**a**) Homogeneous networks, $\langle k\rangle =4$; (**b**) heterogeneous networks, $\langle k\rangle =4$. Setup of other parameters is b=0.08, d=0.05, β=0.175, γ=0.05, δ=0.2, and λ=0.3. The number of infected and recovered individuals at initial time step is I(0)=1 and R(0)=0.

In Section 2, we have examined the presence of the endemic equilibrium point $E_{*}$ in our model when $R_{0}>1$, as illustrated by the simulation results presented in Figure 1. In this model, the involvement of newly born susceptible individuals in epidemic spreading (SPES) leads to the emergence of an endemic equilibrium point, ensuring the perpetuation of the epidemic. Conversely, when newly born susceptibles do not participate in epidemic spreading (SNES), our model will only exhibit a disease-free equilibrium point $E_{0}$. In the context of the Gillespie algorithm, the model exclusively permits the increase or decrease of one infected node within a unit time interval Δt, without considering the additional replenishment of susceptible individuals. As such, in the ensuing numerical simulations, we operate under the assumption that newly added susceptible individuals at each time step do not partake in the transmission of the infectious disease. Instead, following the rules outlined in this paper, these individuals are introduced into our proposed evolutionary model at a fixed proportion. Operating under this assumption, the model manifests a unique disease-free equilibrium point, as depicted in Figure 1. Additionally, it is important to note that, in the model, β=0.175 indicates that susceptible individuals have a 17.5% probability of being infected upon contact with infected individuals, while γ=0.05 denotes a 5% chance per unit time for infected individuals to transition to the recovered state.

In addition, owing to the inclusion of the birth rate for susceptible individuals in our model, it becomes apparent that, during the initial phases of the infectious disease’s evolution, when newly born susceptible individuals participate in disease transmission (SPES), the proportion of S(t) exceeds 1, as illustrated in Figure 1. However, as the infectious disease evolves over time, an increasing number of susceptible individuals gradually transition into infected status. Eventually, a dynamic equilibrium is established between the newly susceptible individuals and those transitioning into the infected states. Notably, the emergence of an endemic equilibrium point signals the accomplishment of this dynamic balance.

### 3.2. Comparison between MCS and CTMC Methods

**Figure 2:**
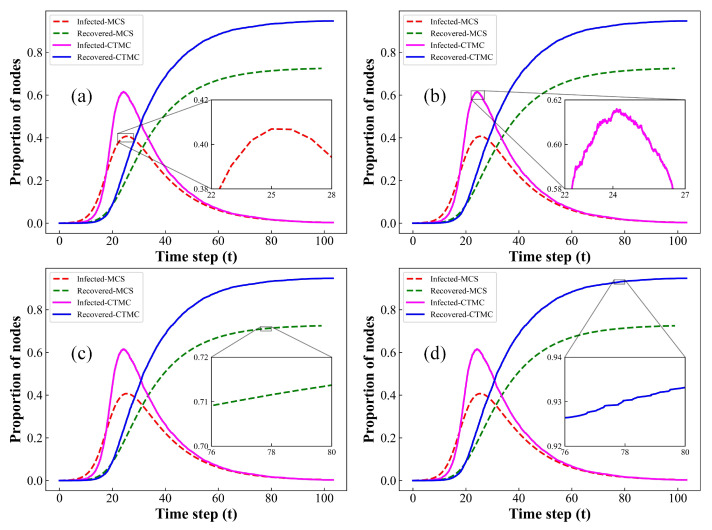
Comparison of the evolution curves of the proportions of I(t) and R(t) based on MCS and CTMC methods at each time step on homogeneous networks with average degree $\langle k\rangle =4$. (**a**) Local amplification of Infected-MCS; (**b**) local amplification of Infected-CTMC; (**c**) local amplification of Recovered-MCS; (**d**) local amplification of Recovered-CTMC. Setup of other parameters is same as Figure 1.

In this subsection, we compare the epidemic propagation curves of our model using the MCS and CTMC methods, respectively. Additionally, since the bidirectional immunization proposed in this model primarily influences the density of infected and recovered individuals, the simulation results are focused solely on the dynamics of I(t) and R(t). Compared to MCS and MF, CTMC accounts for the variation in the number of infected individuals in the system over a time interval Δt shorter than each time step *t*. Therefore, the CTMC model provides a more accurate representation of the infectious disease transmission process in real-world scenarios. In Figure 2, we compare the MCS and CTMC approaches in homogeneous networks. The result illustrates that both simulation methods exhibit similar evolutionary trends. Additionally, similar to the MF approach, the CTMC model assumes that epidemics propagate within a well-mixed framework, freeing the transmission of epidemic diseases from the constraints of network structure and resulting in an optimal transmission effect. Therefore, the CTMC model demonstrates higher infection peaks and a broader transmission range, as illustrated in Figure 2. Moreover, CTMC assumes that, within sufficiently short time intervals Δt, only one of two events can occur: the transition of a susceptible node to an infected node or the transition of an infected node to a recovered node. Thus, the simulation results reveal continuous fluctuations in data over short time periods in the CTMC model.

**Figure 3:**
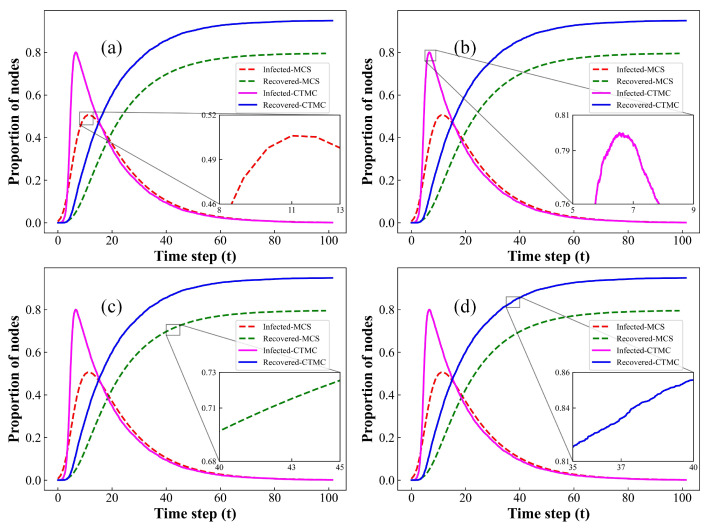
Comparison of the evolution curves for the proportions of I(t) and R(t) through MCS and CTMC methods at each time step on heterogeneous networks with average degree $\langle k\rangle =4$. (**a**) Local amplification of Infected-MCS; (**b**) local amplification of Infected-CTMC; (**c**) local amplification of Recovered-MCS; (**d**) local amplification of Recovered-CTMC. Setup of other parameters is same as Figure 1.

In Figure 3, we compare the simulation results between MCS and CTMC within heterogeneous networks. Clearly, the observations made in Figure 2 persist in the context of heterogeneous networks, as depicted in Figure 3. Notably, in homogeneous networks, the degree distribution closely approximates a Poisson distribution, demonstrating a high degree of uniformity. In contrast, for heterogeneous networks, the degree distribution exhibits a pronounced scale-free feature, closely resembling a power-law distribution. Consequently, within heterogeneous network models, certain nodes exhibit degrees significantly higher than others. Moreover, as illustrated in Figure 3, the rate and extent of disease transmission in heterogeneous networks are significantly higher compared to homogeneous networks. This leads to the conclusion that network heterogeneity, characterized by scale-free properties, can effectively facilitate disease transmission. Hence, in infectious disease control efforts, it is crucial to accurately monitor and control the mobility of those with the highest contact rates and super-spreaders, as they can significantly expedite the spread of epidemics.

### 3.3. Comparison between MF and CTMC Methods

**Figure 4:**
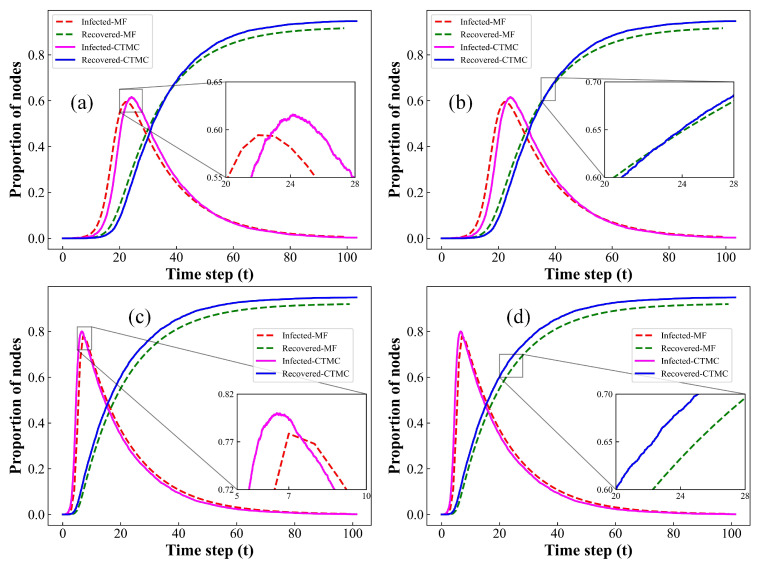
Comparison of the evolution curves for the proportions of I(t) and R(t) through MF and CTMC methods at each time step on homogeneous and heterogeneous networks with average degree $\langle k\rangle =4$. (**a**) Homogeneous networks, local amplification of I(t); (**b**) homogeneous networks, local amplification of R(t); (**c**) heterogeneous networks, local amplification of I(t); (**d**) heterogeneous networks, local amplification of R(t). Setup of other parameters is same as Figure 1.

In this section, we compare the simulation results between the MF and CTMC approaches for both homogeneous and heterogeneous networks. The results are illustrated in Figure 4. Both CTMC and MF models assume that epidemics spread in well-mixed network models, neglecting the influence of network structure on disease transmission. As depicted in Figure 4, the propagation curves of our model, generated by two distinct simulation methods, indeed demonstrate a high degree of similarity. Therefore, the CTMC model effectively reproduces the transmission patterns based on the rules proposed in this paper, closely mirroring the performance of the MF method. Additionally, akin to the simulation outcomes illustrated in Figure 2 and Figure 3, the CTMC-based model also accurately captures the inherent stochastic fluctuations present throughout the transmission of infectious diseases. Furthermore, the substantial agreement observed in the simulation outcomes between the CTMC and MF models indirectly validates the precision of the steady-state distribution and transition probabilities derived from the CTMC model in Section 2.2. These results provide a precise probabilistic depiction of the inherent propagation patterns in our proposed model.

### 3.4. Impact of Bidirectional Immunization on Epidemic Spreading

The present study introduces an enhanced bilinear SIR model that incorporates bidirectional immunization and the birth and death of individuals. In this model, δ represents the immunity rate of susceptible individuals after vaccination while λ represents the recovery rate and formation of immunity post-infection of individuals. In this subsection, our primary objective is to explore the influence of bidirectional immunity (post-infection and post-vaccination) on the spread of infectious diseases within complex networks. We achieve this by analyzing numerical simulation results obtained through the MCS method. To reduce the impact of randomness on simulation outcomes, the results of each set are averaged over $1.0\times 10^{3}$ independent replicate experiments.

**Figure 5:**
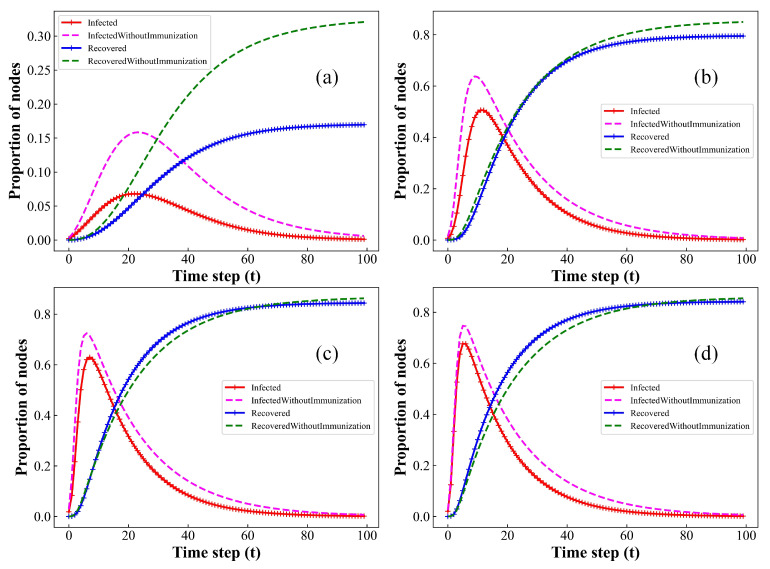
Comparison of the fraction of I(t) and R(t) with and without immunization measures over time, in BA networks with varying average degree $\langle k\rangle$. (**a**) $\langle k\rangle =2$; (**b**) $\langle k\rangle =4$; (**c**) $\langle k\rangle =6$; (**d**) $\langle k\rangle =8$. Setup of other parameters is same as Figure 1. As average degree $\langle k\rangle$ increases, the impact of bidirectional immunization measures on epidemic spreading gradually diminishes.

Initially, as illustrated in Figure 5, the implementation of bidirectional immunization measures results in a reduction in infected individuals and an increase in the number of recovered individuals. However, with the increase in the average degree of the network, the impact of bidirectional immunity measures on infectious disease transmission gradually diminishes until it disappears entirely. This result indicates that the effectiveness of immunization measures diminishes with an increase in the individuals’ contact rates. Hence, following an epidemic outbreak, effective disease control strategies might consider prioritizing the regulation of interactions among individuals in the affected area before implementing immune interventions such as vaccination and clinical treatments.

**Figure 6:**
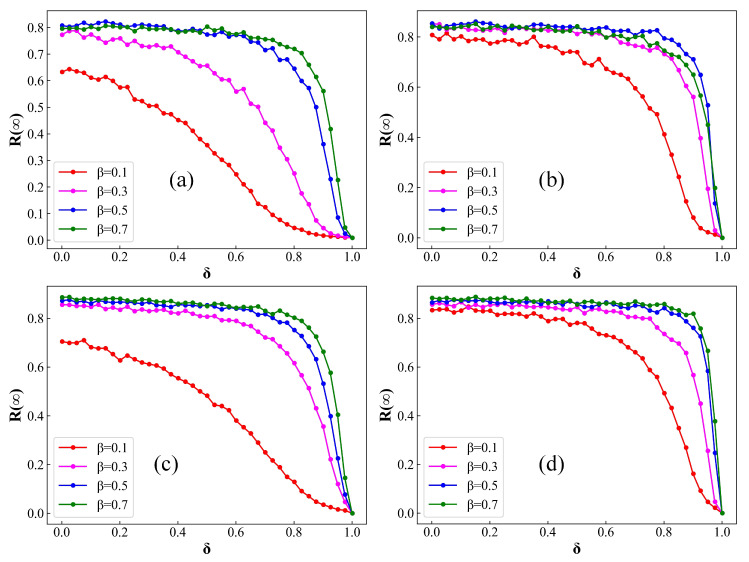
Fraction of R(t) at steady state on ER and BA networks as a function of δ for distinct values of β. (**a**) ER network, $\langle k\rangle =4$; (**b**) ER network, $\langle k\rangle =8$; (**c**) BA network, $\langle k\rangle =4$; (**d**) BA network, $\langle k\rangle =8$. Setup of other parameters is same as Figure 1. An augmentation in the parameter δ leads to a discernible decrease in the magnitude of epidemic propagation in both ER and BA networks.

**Figure 7:**
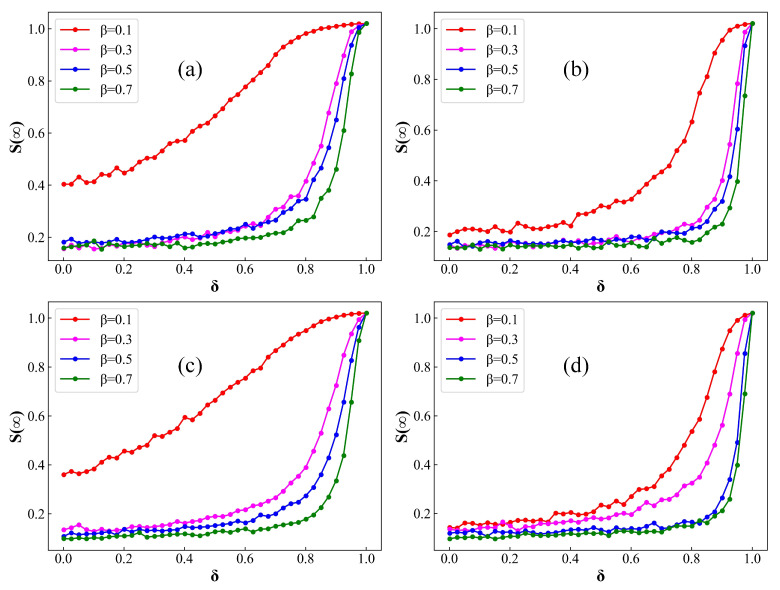
Fraction of S(t) at steady state on ER and BA networks as a function of δ for distinct values of β. (**a**) ER network, $\langle k\rangle =4$; (**b**) ER network, $\langle k\rangle =8$; (**c**) BA network, $\langle k\rangle =4$; (**d**) BA network, $\langle k\rangle =8$. Setup of other parameters is same as Figure 1. With the incremental rise in δ, there is a notable augmentation in the magnitude of S(∞).

In Figure 6 and Figure 7, we systematically examine the correlation between δ and the steady-state values of both R(∞) and S(∞) within ER and BA networks. Herein, R(∞) and S(∞) denote the proportion of compartments R(t) and S(t), respectively, when the system reaches its equilibrium state. In particular, it is noteworthy that the impact of random numbers in Monte Carlo simulations leads to observable random fluctuations in the experimental results. In Figure 6, with an increase in the distribution of the average number of contacts between an infected individual with a susceptible individual, the extent of disease transmission in both ER and BA networks exhibits varying degrees of growth. This highlights that elevated levels of population contact undeniably contribute to the facilitated spread of infectious diseases, aligning with prior research findings. Additionally, under identical immunization rate conditions, the steady-state fraction of infected individuals, denoted as R(∞), increases with the escalation of the infection rate β. This outcome suggests that the intrinsic transmission capacity of the infectious disease plays a decisive role in determining the scale of infection. Moreover, with an increase in the immunization rate, denoted by δ, there is a noteworthy decrease in the value of R(∞). As the immunization rate approaches 1, the disease tends to fade away. This underscores the critical importance of immunizing susceptible individuals as a pivotal measure in controlling the spread of infectious diseases.

In Figure 7, as β increases, the proportion of zero-infected individuals gradually diminishes. The simulation results indicate that, in the absence of immunization interventions, controlling individual contacts and gatherings can effectively suppress the transmission of less infectious epidemics but proves ineffective against highly contagious diseases. Additionally, when $\langle k\rangle$ is set to 8, in the absence of immune intervention measures, the density of S(∞) approaches zero. This result suggests that, under conditions of a sufficiently high population contact rate, the containment of epidemic spread relies exclusively on the implementation of immunization measures directed towards susceptible individuals. Moreover, Figure 7 also reveals that, as vaccination coverage δ increases, the proportion of susceptible never-infected individuals also increases. This denotes that the effectiveness of epidemic prevention is positively correlated with the vaccination rate among susceptible individuals, aligning with the conclusion drawn from Figure 6. Therefore, attaining the maximum vaccination rate among susceptible individuals with the highest contact rates emerges as the most effective strategy for both controlling and ultimately eliminating infectious diseases.

**Figure 8:**
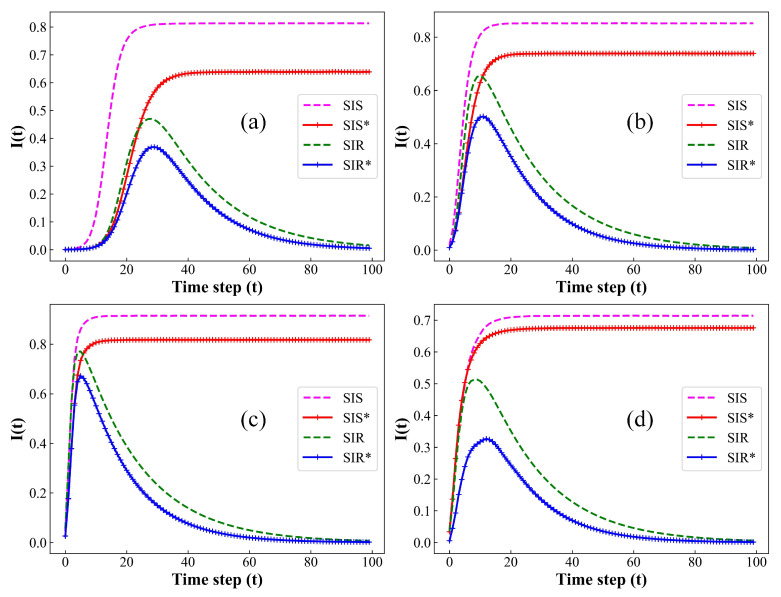
Fraction of I(t) at each time step on different types of network datasets under various propagation models. (**a**) ER network, $\langle k\rangle =4$; (**b**) BA network, $\langle k\rangle =4$; (**c**) Facebook social network, $\langle k\rangle =43.69$; (**d**) Eneon mail communication network, $\langle k\rangle =10.02$. Setup of other parameters is same as Figure 1. In various datasets, bidirectional immunization measures can effectively reduce the infection peaks and steady-state infection density of epidemics.

Subsequently, we will conduct a comparative simulation analysis of our model across four network datasets: ER networks, BA networks, the Facebook social network, and the Eneon email communication network. The ER and BA networks are synthetic, while the Facebook social network and the Eneon email communication network are real-world datasets [34,35]. Specifically, the Facebook social network comprises 4039 nodes (susceptible individuals) and 88,234 edges (contacts to susceptible individuals or number of susceptible individuals with a contact to an infected individual) with an average degree of 43.69 (number of susceptible person contacts for an infected individual); the Eneon email communication network includes 36,692 nodes and 183,831 edges, with an average degree of 10.02. Moreover, we have extended the SIS model by introducing bidirectional immunization, as well as birth and death rates, and conducted comparative experiments with the traditional SIS and SIR models. Specifically, we assume that the infection rate and recovery rate of the SIS model are consistent with the SIR model. The remaining parameters of the model are consistent with those in Figure 1 and the simulation results are illustrated in Figure 8, where SIR* represents the transmission model proposed in this study and SIS* represents the SIS model extended with bidirectional immunization, as well as birth and death rates. Differently from the SIR model, in the SIS model, infected individuals have a certain probability of reverting to susceptible states. Therefore, in the simulation results, the evolution curve of the infected individual density, I(t), over each time step *t* in the SIS model exhibits the steady-state equilibrium point. In Figure 8, it can be observed that the immunization effectiveness of the improved model is superior in ER networks compared to BA networks, indicating that the heterogeneity of the network exerts an inhibitory effect on immunization measures. Furthermore, for the same propagation model, the steady-state density and transmission peak of I(t) are significantly higher in the smaller Facebook network with four times higher average degree (number of contacts) compared to the larger-scale Eneon network. Therefore, in comparison to the network’s node scale, the average degree of the population contact network exerts a more pronounced impact on the infection scale of the infectious diseases. Additionally, our simulation results confirmed that, when contrasted with the standard epidemic model, the enhanced bidirectional immunity model exhibits lower infection peaks by 20%, a diminished steady-state infection density by 30%, and a decelerated transmission speed. Furthermore, this conclusion remains valid in both theoretical datasets and real large-scale network datasets. Thus, this provides further confirmation that bidirectional immune effects effectively reduce the scale of epidemic transmission.

### 3.5. Sensitivity Analysis of Basic Reproduction Numbers

In the field of infectious disease dynamics, the basic reproduction number is a critical concept. It signifies the number of susceptible individuals that an infected individual can infect during their infectious period, thus characterizing the potential for disease spread. Furthermore, the basic reproduction number is also a vital indicator for determining whether an infectious disease will become an epidemic. Typically, when $R_{0}$ is greater than 1, an epidemic outbreak occurs; conversely, if $R_{0}$ is less than or equal to 1, the disease gradually fades away. In this subsection, we analyze the sensitivity of the basic reproduction number to various parameters in both homogeneous and heterogeneous networks.

**Figure 9:**
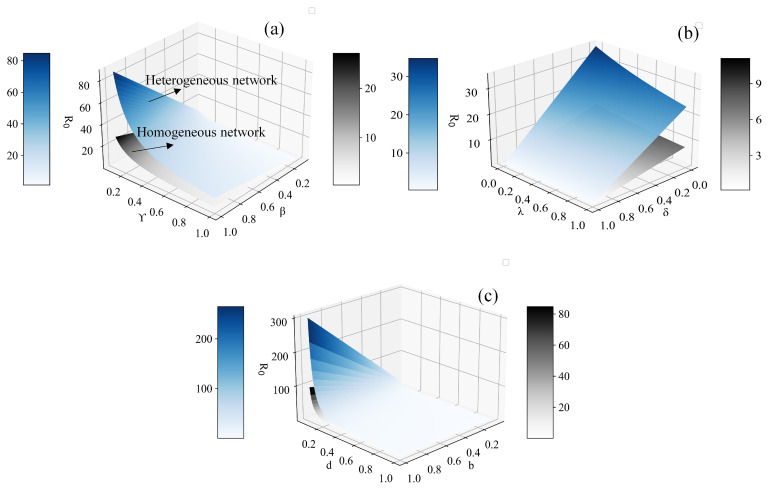
Comparison of the basic reproduction number $R_{0}$ in homogeneous and heterogeneous networks: impact of epidemic spreading parameters, bidirectional immunization rates and the rates of birth and death. (**a**) Variations in β and γ, $\langle k\rangle =4$; (**b**) variations in δ and λ, $\langle k\rangle =4$; (**c**) variations in *b* and *d*, $\langle k\rangle =4$. Setup of other parameters is same as Figure 1.

In Figure 9, we analyze the impact of three parameter pairs on the basic reproduction number. These pairs consist of birth rate *b* and death rate *d*, infection rate β and recovery rate γ, and immunity rate for the infected individuals δ and immunity rate for the recovered individuals λ. The simulation results reveal that, compared to the immunity rates δ and λ, $R_{0}$ is more sensitive to β and γ, indicating that the inherent infection and recovery rates of the infectious disease are the primary influencing factors on its transmission capability. Additionally, as depicted in Figure 9b, variations in λ have a comparatively minor impact on the value of $R_{0}$ when contrasted with changes in δ. This result indicates that immunization measures of susceptible individuals are more effective in reducing the transmission capacity of infectious diseases compared to immunity due to natural infections as those individuals infect more individuals before becoming immune. Furthermore, Figure 9c indicates that extreme values of the death rates have a significant impact on $R_{0}$, which is determined by the mathematical properties of the basic reproduction number. In addition, the values of *b* and *d* within their normal ranges do not substantially affect the value of $R_{0}$, suggesting that the inflow and outflow of individuals within a certain acceptable range have limited influence on the transmission capacity of epidemics. While this finding suggests that imposing stringent restrictions on population mobility in affected areas may not lead to significant changes in population numbers, it is well established that population mobility often substantially increases contact rates, serving as a well-established mechanism for introducing infectious diseases to immunologically naive populations. Hence, without disrupting the regular activities of ordinary citizens, minimizing population movement in epidemic zones and reducing interpersonal contact frequency can be viewed as effective measures to curb the spread of the epidemics. Additionally, Regardless of parameter values, $R_{0}$ consistently exceeds that in heterogeneous networks compared to homogeneous networks, indicating a more pronounced facilitating effect of heterogeneity on infectious disease transmission, aligning with the earlier discoveries.

## 4. Conclusions and Discussion

In this paper, we propose an extended network-based SIR model that takes into account bidirectional immunity as well as the birth and death rates of individuals. We conduct theoretical analyses of this model, calculating the basic reproduction numbers and corresponding epidemic thresholds by examining the conditions for the existence of endemic equilibrium points in both homogeneous and heterogeneous networks. Subsequently, we utilize CTMC to formulate our proposed model, examining the steady-state distribution and transition probabilities of the CTMC-based epidemic model in homogeneous and heterogeneous networks, respectively. Furthermore, the transition rates matrix based on the embedded chain for our proposed propagation pattern is also derived.

Following that, we validate the relevant properties of the model through numerical simulations. Considering the characteristics of the MCS and CTMC simulation algorithms, in our experimental simulations, we operate under the assumption that newly born susceptible individuals do not immediately participate in disease transmission. Therefore, the results of the simulations do not indicate the presence of an endemic equilibrium point. Additionally, simulation results also indicate that, in comparison to the network-based MCS method, epidemics spread more extensively in the CTMC and MF methods, which assume a well-mixed model for disease transmission. The results demonstrate that the well-mixed model, established on the basis of the mean-field hypothesis, represents an ideal scenario for infectious disease transmission. Furthermore, while the simulation data from the CTMC approach displays some fluctuation, the results of the simulations also demonstrate a good fit between the CTMC and MF models. The analysis presented in Section 2.2 offers theoretical foundation for the presence of these stochastic fluctuations. In the CTMC model, it is assumed that, within a sufficiently small time interval Δt, only one event occurs, representing either an increase in infected nodes (indicating susceptible nodes becoming infected) or a decrease in infected nodes (indicating infected nodes transitioning to the recovered state). The pair of susceptible and infected node quantities, represented as a tuple $\left(s(t),i(t)\right)$, constitutes the fundamental state space set of the continuous-time Markov chain. Within our model, state transitions occur between adjacent state spaces, facilitating the progression of infectious disease transmission. Significantly, the incorporation of a probabilistic perspective leads to continuous stochastic fluctuations in the simulation results of the CTMC model over brief time intervals. This characteristic enriches the CTMC model’s portrayal of the dynamics of infectious diseases, aligning it more closely with real-world scenarios.

Additionally, we utilize MCS to assess the effects of bidirectional immunization measures on the transmission of infectious diseases in networks. The results indicate that, as the network’s average degree increases, the effectiveness of the immunization measures gradually diminishes. This observation suggests that the anticipated effectiveness of immunization measures can only be realized when population contact rates are sufficiently low. Hence, it is crucial to reduce interactions among the population in an epidemic area before implementing immunity measures for individuals. Moreover, the relationship between the steady-state infection density and the immunization rate of susceptible individuals is also the focus of our study. The experimental results indicate a notable decrease in the steady-state infection density of the infectious disease as the immunization rate increases. This suggests that the implementation of robust immunization measures targeting susceptible individuals can effectively control the spread of the epidemics.

Finally, we explore how three distinct sets of pivotal parameters within this model impact the value of the basic reproduction number. The simulation results emphasize the pivotal role of infection and recovery rates in determining the transmission capacity of the infectious disease. In comparison, immunization measures targeting susceptible individuals have a more pronounced effect on the basic reproduction number compared to clinical treatment measures directed at recovered individuals. This highlights the effectiveness of post-outbreak immunization measures aimed at susceptible individuals in managing the extent of infectious diseases spread.

In our research, besides analyzing the proposed model using conventional mean-field methods, we endeavored to map the transmission dynamics of infectious diseases onto a comprehensive CTMC framework. This approach offered an additional analytical perspective, employing probabilistic methods and contributing to a certain degree of theoretical innovation. Within the CTMC framework, when the time intervals between state transitions are sufficiently small, state changes can be treated as continuous. Conversely, the Gillespie algorithm discretely updates states at each event occurrence, providing an approximation of continuous-time processes. Through careful selection of appropriate time steps, the discretized process can effectively mimic the continuous-time evolution. Furthermore, the integration of discrete-time reasoning and a probabilistic viewpoint in the CTMC method enables the observation of stochastic fluctuations and oscillations in individual density over time within the simulated results. This feature enhances the model’s fidelity to real-world scenarios of disease spread. However, beyond the mere existence of endemic equilibrium points, the analysis of the stability of both disease-free and endemic equilibrium points constitutes a crucial aspect of infectious disease dynamics research [36,37,38,39,40,41,42,43,44,45,46]. Accordingly, our forthcoming research will primarily focus on examining the stability of equilibrium points within infectious disease models, thereby contributing significantly to the advancement of our work.

## Data Availability

No new data were created or analyzed in this study. Data sharing is not applicable to this article.
